# Supplementary material for: COVID-19: Retransmission of official communications in an emerging pandemic
Source: PLoS One. 2020 Sep 16;15(9):e0238491. doi: 10.1371/journal.pone.0238491 (PMC7494104; doi:10.1371/journal.pone.0238491)
Supplement: Attachment — Submitted filename: Response to Reviewers.docx [file pone.0238491.s002.docx]

Response to Reviewers – “COVID-19: Retransmission of Official Communications in an Emerging Pandemic”

Style guide –

Table 1 – we have removed the images from the table and have replaced them with fictitious examples that illustrate the kind of images that exist in our data.

ORCID iD for corresponding author. 0000-0002-4345-9108

The paper has been formatted per PLOS One style guide.

**Reviewer 1:**

A suggestion - When measuring period effects, it is more useful to divide the time of the day to morning, afternoon, night... Splitting it on the hourly basis is too fine-grained and can result in smaller sample sizes and the lower confidences.

*We offer a response to this suggestion in the methods section (line 479), explaining that “the large volume of available messages allows us to reliably estimate daily cycles at hourly resolution, as can be seen from the standard errors in Table 3.*

**Reviewer 2:**

I recommend including a visualization of the retweet distribution to demonstrate its skew. Using a log-log scale would help with this.

*We have provided an additional visualization (figure 2) to demonstrate retweet distribution as requested.*

- I'd like to see the formula for the negative binomial regression model written somewhere.

*We now include the formula for the negative binomial regression model in our methods section.*

- In the first few paragraphs of the discussion, the several references to prior research make it somewhat difficult to tell which statements are being attributed to your original work. Some simple phrasing (eg. "we find that...") can help with this.

*Thank you, for this suggestion. We have revised the discussion section to make this clearer.*

- In the conclusion, it feels like a reach to attribute lower likelihoods to exclamatory and interrogative "language", given that this part of the analysis was entirely based on punctuation.

*Thank you for this suggestion. We have addressed this concern by noting that we are analyzing sentence styles that use exclamatory and interrogative punctuation.*
